# Supplementary material for: New predictive model of the touchdown times in a high level 110 m hurdles
Source: PLoS One. 2022 Dec 2;17(12):e0278651. doi: 10.1371/journal.pone.0278651 (PMC9718393; doi:10.1371/journal.pone.0278651)
Supplement: Attachment — Submitted filename: Response to Reviewer2.docx [file pone.0278651.s003.docx]

Response to Reviewer 2:

The authors would like to thank the reviewer for their valuable comments. We are delighted to hear that you think our work will spark debate in our field. In the following sections, you will find our responses to each of your points and suggestions.

1. [This paper presents a revised prediction model for the 110 m sprint hurdles event based on a cross validated regression model. The paper is technically sound and the conclusions are supported by the data. The writing is generally clear and intelligible, however the structure of the paper does not adhere to reporting guidelines and major revisions are required in restructuring. In particular, results appear in the discussion and method related information appears in the in the results. These shortcomings can be rectified but will require extensive revision. Overall the topic has merit and will be of value to sports scientists and practitioners. The authors repeatedly claim that this is a novel method of analysis however, LOOCV is a well established technique except that it has not been applied to these data. I advise the claim of novel methods should be omitted as this is not accurate and it will not detract from the originality of the study to remove the claim of novel methods.]

Thank you for your valuable feedback. We have revised the structure of the paper. As you stated, LOOCV is not a novel method. We have removed “novel” to explain LOOCV from the paper.

SPECIFIC COMMENTS:

2. [P 2 ln 18: I don't think the methods used in this model are novel. The regression and cross validation techniques used in this study has been used for many years.]

Thank you for your advice. We have changed “novel” to “robust, reliable” (lines 19, 33, 86).

3. [P 2 ln 19: to predict performance based on touchdown times.]

We have revised it (line 21).

4. [P2 ln R2 should be R (2 superscript)]

We have revised it (30, 31).

5. [P3 ln 39: "time of interval running" verbose and unclear. Hurdle touchdown period or intervals is easier to understand]

Thank you for your suggestion. We have changed “interval times”. We also added “between hurdles” because Reviewer 1 pointed out the definition of intervals (line 46).

6. [P3 ln 44: There are various verbose expression that appear throughout for example "to make the interval running time shorter" ... perhaps " to reduce touchdown periods". Please do a thorough recheck and revise.]

We have rephrased verbose expressions (line 50).

7. [P3 Ln 46: Spatiotemporal variables is vague, perhaps just be specific ... you only used hurdle touchdown times.]

Thank you for pointing this out. As you stated, we only used hurdle touchdown times. We have revised as shown (lines 55–57): “In the hurdle events, times from the start signal to the instants of the leading leg landing after clearing the 1st to 10th hurdles (touchdown time) are typical parameters.”

8. [P3 ln 60: "To the best of our knowledge" perhaps avoid this expression ... "The model appears to be based only on...."]

We have revised as shown (line 69): “To date, the model appears to be the only one based on a statistical analysis of actual races of the 110-m hurdles.”

9. [P4 ln 66: "it is quite unlikely to apply the model to predict temporal parameters" poor grammar, please revise.]

In accordance with the comment, we have revised it (lines 73–75).

10. [P4 ln 67 "evaluated the prediction accuracy of the regression model using the training data", Training data in the context of the study could be confusing here since training data could be data derived out of competition or it refers to training data sets for the regression model. please try to be clear.]

We agree with your opinion. We tried to avoid confusion by referring to that training data in this context means the data used in the regression model (lines 78–79).

11. [P4 ln 84: Please clarify whether these data were based only on competition data and if not why data from a training situation were not removed.]

Thank you for pointing this out. These data were based only on competition data. We have added this fact (lines 96–97).

12. [P8 ln 152: "novel statistical model" this is not a novel approach]

We have changed “novel” to “a more representative” (line 208).

13. [P8 ln 155 "better predictable performance" perhaps Better prediction performance"]

We have revised it (line 211).

14. [P9 ln 175-185: this information is out of sequence as it is methods information. Please follow standard structure]

In accordance with the comment, the relevant parts have been described in the methods.

15. [P9 ln 178: This is a misuse of correlation and significance. The fact that a correlation is not statistically significant does not mean that RT had no effect. To illustrate this if two samples are compared and the t test finds no statistically significant difference does not mean that the mean scores are equal. Therefore you cannot dismiss the impact of RT on performance by reporting that the correlation of RT with final performance in not significant. Later this idea is rectified but you should not imply the lack of significance means no difference or no effect.]

You have raised an important point. I appreciate your comment. We were simply interested in how the reaction time affects the predictive performance of the model. Therefore, we have removed this sentence because we think that misleading here should be avoided.

16. [P10 ln 196 This table presents results and should appear in the results section not the discussion]

The relevant parts have been described in the results.

17. [P11 ln 216-221: this is Methods]

The relevant parts have been described in the methods.

18. [P12 ln 234: The table presents results and should be in the results section.]

The relevant parts have been described in the results.

19. [Major restructuring of the results and discussion sections are required.]

Thank you for pointing this out. We have modified the structure of the paper.
