# Supplementary material for: New predictive model of the touchdown times in a high level 110 m hurdles
Source: PLoS One. 2022 Dec 2;17(12):e0278651. doi: 10.1371/journal.pone.0278651 (PMC9718393; doi:10.1371/journal.pone.0278651)
Supplement: Attachment — Submitted filename: Response to Reviewer2.docx [file pone.0278651.s004.docx]

Response to Reviewer 2:

The authors would like to thank the reviewer for their valuable comments. We are delighted to hear that you think our work will spark debate in our field. In the following sections, you will find our responses to each of your points and suggestions.

1. [Thank you for your revised manuscript. In general the revisions have addressed the technical issues I identified in the previous review, however there remain several grammatical and expression issues throughout the revised paper. Some of these grammatical errors appear in the revised sections while other errors appear in the previous text. I have included details of specific revisions required but a detailed proof check of the manuscript is still required.]

Thank you for pointing that out. We carefully checked the grammatical and expression issues of the manuscript. There are several revisions in this regard (Ln 20, 50, 57, 75, 83, 96, 98, 104, 204, 214, 245, 268). Our responses to each point are the following.

2. [Ln 20 : perhaps elite rather than elite-standard throughout. Also you need to be consistent in the format of number unit especially with respect to distances we have 110-m, 1.067 m appearing best to have one format consistently I suggest 110 m not 110-m]

Thank you for your suggestion. We have revised it (Ln3, 20, 38, 39, 40, 41, 60, 66, 68, 82, 204, 210, 214, 261, 267, 271, 272). A hyphen in the title was also excluded for consistency of expression.

3. [Ln 33-37: suggest you use “this study” rather than "the present study”]

Thank you for your suggestion. We have revised it (Ln 35). We have also revised several in this regard (Ln 85, 209, 260, 263).

4. [Ln 36: “….would help to improve training interventions…..”]

Thank you for your suggestion. We have revised it (Ln 36).

5. [Ln 44: replace “namely” with “by”]

We have revised it (Ln 43).

6. [Ln 46: reconsider: significantly correlated (r = 0.81–0.99); perhaps, “strongly correlated (r = 0.81–0.99; p<0.05)”]

We have revised it (Ln 45).

7. [Ln53-54: Are spatiotemporal variables important determinants of performance rather than just “important for better performance”?]

Thank you for pointing this out. As you stated, spatiotemporal variables are important determinants of race outcomes. We have rephrased it (Ln 51).

8. [Ln 59: replace “To do so, this is necessary“ with “this is necessary”.]

We have revised it (Ln 57).

9. [Ln 66-67: the grammar in this sentence is poor and therefore expression is unclear. Please revise.]

We have revised it (Ln 66–67).

10. [Ln 73-75: “Thus, it is reasonable to interpret that the existing model is inapplicable to predicting…” this expression is verbose, please simplify (I suggest: “The existing model therefore fails to reliably predict the touchdown times…..”)]

Thank you for your suggestion. In accordance with your suggestion, we have revised it (Ln 71–72).

11. [Ln 75-77: Moreover…..prediction accuracy”. This new sentence is poorly constructed and it does not add anything of value to the text, I suggest you omit it.]

In accordance with the comment, we have omitted it.

12. [Ln 83 – 86: In my earlier review, I suggested the need to strengthen the rationale for the study or make the rationale more obvious. This can be done by simply stating that based on the previous discussion, it is clear that the existing model does not provide accurate predictions, therefore a more robust and reliable model is required. Your text in this section is verbose and misses this simple statement of need.]

Thank you for pointing this out. As you stated, our text was verbose and unclear. We have revised it (Ln 78–81).

13. [Ln 97 omit "actual"]

We have omitted it.

14. [Ln 137-138 try “ a numerical experiment for net touchdown times (without the reaction time) was completed.]

We have revised it (Ln 133).

15. [Ln 139: try “touchdown times with reported reaction times”]

We have revised it (Ln 133–134).

16. [Ln 233 omit possess]

We have omitted it.

17. [Ln 268: revise : “The model also allows…”]

We have revised it (Ln 263).
